# iGABASnFr Imaging Reveals that GABA Clearance Is Rapidly Overwhelmed in the Cerebral Cortex

**DOI:** 10.1523/JNEUROSCI.0354-26.2026

**Published:** 2026-10-14

**Authors:** Reyna L. Gariepy, Jesse B. Blackman, Anushree S. Gupte, Jeffrey S. Diamond, Chris G. Dulla, Moritz Armbruster

**Affiliations:** ^1^Department of Neuroscience, Tufts University School of Medicine, Boston, Massachusetts 02111; ^2^Neuroscience Program, Tufts Graduate School of Biomedical Sciences, Boston, Massachusetts 02111; ^3^Synaptic Physiology Section, NINDS Intramural Research Program, National Institutes of Health, Bethesda, Maryland 20892

**Keywords:** GABA, GAT, iGABASnFr, inhibition

## Abstract

Both phasic and tonic GABAergic activities in the cortex are shaped by the clearance of GABA from the extracellular space by GABA transporters (GATs). However, estimates of the time-course of GAT activity from GAT currents, GABA_A_, and GABA_B_ electrophysiological recordings range from tens of milliseconds to multiple seconds. These estimates may reflect receptor properties rather than extracellular GABA dynamics. Understanding how long GABA is free in the extracellular space is key to understanding how inhibition controls activity but has been technically challenging. Here, we utilize the fluorescent GABA sensor, iGABASnFr, to enable us to optically assay extracellular GABA dynamics following bursts of synaptic activity. In the mouse cerebral cortex of either sex, we show that GABA rapidly disperses from release sites via diffusion, with very little role for active transport by GATs. Following diffusion from the sites of release, GABA then accumulates in the extracellular space, where GATs require hundreds of milliseconds to remove it. For areas of high-density GABA release or during bursts of activity, this clearance is further slowed. Continued GABA release and summation in the extracellular space for bursts of activity contrasts with recorded inhibitory postsynaptic current (IPSC) signals, which rapidly depress with additional stimuli, suggesting a disconnect between IPSCs and extracellular GABA. Lastly, the resulting extracellular accumulation of GABA can act heterosynaptically by activating and/or desensitizing GABA_A_ receptors. Together, this raises new questions about how GABAergic inhibition works to shape network function and suggests a spatiotemporal continuum of extracellular GABA.

## Significance Statement

We use combined iGABASnFr neurotransmitter imaging, electrophysiology, and modeling to show that with trains of synaptic activity, GABA clearance is slowed and GABA rapidly accumulates in the extracellular space. Additionally, we demonstrate that GABA largely diffuses from release sites rather than being captured by GABA transporters and that these transporters have only a small role in shaping extracellular GABA dynamics in the mouse cortex. Lastly, we show that extracellular GABA accumulation can heterosynaptically desensitize GABA_A_ receptors, modulating local phasic inhibition.

## Introduction

GABAergic inhibition powerfully constrains brain function, enabling the precise timing of neuronal activity, synchronizing systems across different timescales, and preventing aberrant excitation. Upon vesicle release, GABA in the synaptic cleft reaches millimolar concentrations, sufficient to activate fast, phasic low-affinity GABA_A_ receptors (GABA_A_Rs), after which it rapidly diffuses out of the synaptic cleft (<1 ms; Quick, 2002). Once outside the synaptic cleft, GABA can activate slow, tonic extrasynaptic GABA receptors (GABAR), metabotropic GABA_B_Rs, or be transported by GABA transporters (GATs; Keros and Hablitz, 2005; Terunuma, 2018; Bhatt et al., 2023). In the cortex, both GAT1 (primarily presynaptic on interneurons) and GAT3 (astrocytic) are expressed (Scimemi, 2014). GATs control GABAR activation and function, and loss-of-function mutations cause epilepsy and ataxia (Chiu et al., 2005; Carvill et al., 2015; Chung et al., 2025). However, pharmacological inhibition of GAT1 with tiagabine is also used as an antiseizure medication (Calcagnotto et al., 2002; Mortensen et al., 2024). GATs are expressed at far lower levels than glutamate transporters (Lehre and Danbolt, 1998; Chiu et al., 2002), suggesting that GABA signaling may not be as robustly constrained by active transport as glutamate (Diamond, 2005). Estimates of the timescale over which GATs are active following synaptic release differ across multiple orders of magnitude, depending on the assay, from 10 s of milliseconds to seconds.

Synaptically evoked GAT currents, recorded from astrocytes (Kinney and Spain, 2002), show that GAT activity persists for 100 s of milliseconds following a single stimulation in the neocortex and is further prolonged following bursts of stimuli. Unique slow inhibitory postsynaptic currents (IPSCs; lasting up to 100 ms) have been recorded in response to volumetric GABA release from neurogliaform interneurons and are sensitive to GAT inhibition (Banks et al., 2000; Szabadics et al., 2007; Karayannis et al., 2010). Measurements of GABA_B_ IPSCs (Scanziani, 2000; Beenhakker and Huguenard, 2010) and presynaptic effects of GABA_B_ (Isaacson et al., 1993; Dittman and Regehr, 1997) also suggest GABA remains in the extracellular space for prolonged periods following release.

The kinetics of phasic GABA_A_-mediated IPSCs (decay ∼20–30 ms) are thought to be driven by GABA_A_ deactivation, desensitization, and diffusion of GABA from the synaptic cleft (Quick, 2002) rather than by GAT-mediated uptake. This is evidenced by miniature and spontaneous IPSCs being unaffected by pharmacological inhibition or genetic deletion of GATs. Stimulus-evoked IPSCs, on the other hand, are prolonged by GAT inhibition, suggesting rapid GAT function. This suggests that even a single stimulus can lead to GABA accumulation in the extracellular space, where GATs can mediate uptake (Rossi and Hamann, 1998; Calcagnotto et al., 2002; Overstreet and Westbrook, 2003; Keros and Hablitz, 2005). Both phasic IPSC recordings (Overstreet and Westbrook, 2003) and GAT currents (Kinney and Spain, 2002) show prolonged decay times with increasing stimulus number and/or intensity, despite operating on dramatically different timescales. This suggests that GABA spillover and summation from multiple synapses drive sensitivity to GATs and argues that GATs function largely outside the synaptic space. Varied estimates of GABA persistence across multiple assays suggest that we do not fully understand extracellular GABA dynamics or how they scale with neuronal activity. Our lack of understanding of extracellular GABA dynamics represents a significant challenge to decoding inhibitory signaling and related dysfunctions (Paulsen and Moser, 1998; Tremblay et al., 2016).

Recent advances allow imaging of GABA using the GFP-based genetically encoded fluorescent GABA sensor, iGABASnFr (Marvin et al., 2019; Kolb et al., 2025). Using iGABASnFr epifluorescence imaging, which reports spatially averaged GABA in the extracellular space, we report that following synaptic stimulation, GABA persists extracellularly for 100's of milliseconds. Using modeling and confocal imaging, we show that GABA rapidly diffuses from sites of release and accumulates in the extracellular space. Lastly, we show that this accumulation can drive heterosynaptic suppression of GABAergic IPSCs. Taken together, these findings indicate that stimulus-evoked phasic GABA release is rapidly transformed into a tonic, volumetric signal due to rapid diffusion and relatively slow GAT-mediated uptake.

## Materials and Methods

### Animals

All procedures were approved by the Tufts University Institutional Animal Care and Use Committee. Male and female C57/Bl6 mice were bred in-house from mice originally obtained from Jackson Laboratory (Strain: 000664), kept on a 12 h light/dark cycle with access to food and water *ad libitum*.

### Adeno-associated virus injection

Somatosensory cortical expression of the iGABASnFr1 or iGABASnFr2 was attained via focal viral injection of iGABASnFr1 (Marvin et al., 2019; AAV1.hSyn.iGABASnFR; Addgene, 112159) or iGABASnFr2 (Kolb et al., 2025; AAV.php.eB.hSyn.iGABASnFR2-WPRE, Virovek). For iGluSnFr imaging, AAV5-hSyn-iGluSnFr (Marvin et al., 2013; University of Pennsylvania Vector Core; catalog #AV-5-PV2723, AV-5-PV2914) was injected as previously described (Armbruster et al., 2016). C57/BL6 mice were stereotactically injected with three injection sites in a single hemisphere (coordinates): (1.25, 1.25, 0.5), (1.25, 2.25, 0.5), and (1.25, 3.25, 0.5) (*λ* + *x*, +*y*, −*z*) mm; 1 µl volume was injected per site with ∼5E9 vector genomes. Following injection, mice were monitored closely for 3 d to ensure recovery. Acute brain slices were prepared for imaging 2–4 weeks following injection. We would like to thank Dr. Loren Looger and the GENIE Project for making the plasmids and viruses available that were used in this study.

### Preparations of acute brain slices

Acute brain slices were prepared as described previously (Armbruster et al., 2016). Mice were anesthetized with isoflurane and decapitated, and the brains were rapidly removed and placed in ice-cold slicing solution containing (in mM) 2.5 KCl, 1.25 NaH_2_PO_4_, 10 MgSO_4_, 0.5 CaCl_2_, 11 glucose, 234 sucrose, and 26 NaHCO_3_ and equilibrated with 95 O_2_:5% CO_2_. The brain was glued to a Vibratome VT1200S (Leica Microsystems), and slices (400 μm thick) were cut in a coronal orientation. Slices were then placed into a recovery chamber containing aCSF comprising (in mM) 126 NaCl, 2.5 KCl, 1.25 NaH_2_PO_4_, 1 MgSO_4_, 2 CaCl_2_, 10 glucose, and 26 NaHCO_3_ (equilibrated with 95 O_2_:5% CO_2_). Slices were allowed to equilibrate in aCSF at 32°C for 1 h.

### Live-slice imaging

Slices were placed in a submersion chamber (Siskiyou), held in place with small gold wires, and perfused with aCSF containing 20 µM DNQX (antagonist of AMPA receptors, Sigma-Aldrich), and 50 µM APV (antagonist of NMDA receptors, Tocris Bioscience), equilibrated with 95 O_2_:5% CO_2_ and circulated at 2 ml/min at 34°C. A tungsten concentric bipolar stimulating electrode (FHC) was placed in the deep cortical layers, and stimulus intensity was set to twice the stimulus required to detect an evoked optical GABASnFR signal. iGABASnFr1 responses were imaged with a 10× water-immersion objective (Olympus) on a custom Olympus microscope with 470 nm LED illumination (Thorlabs). Responses were captured with an Andor Zyla 5.5 (Andor) camera, 12 bit digitization, and 10 ms rolling shutter mode for 100 Hz temporal resolution controlled by MicroManager (Edelstein et al., 2014) in epifluorescence mode using a GFP filter cube (Chroma). Stimulus responses were imaged in sequence: 1 stimulus, 5 stimuli at 100 Hz, 10 stimuli at 100 Hz, and a no-stimulus bleaching control with 10 s between acquisitions. This sequence was repeated 10 times for each slice. For each stimulus, a 3 s imaging window was acquired. Drugs to inhibit GAT1 (25 μM NO-711, Abcam ac120364) and GAT3 (50 μM SNAP-5114, Sigma-Aldrich S1069) were applied for 5 min before recommencing imaging. The specificity of the GABASnFR response was confirmed using the nonbinding version of the iGABASnFr2 sensor, which lacked the stimulus-evoked response (Fig. S1).

For confocal imaging and deconvolution, we used the iGABASnFr2 sensor, which has increased affinity and improved sensor performance (Kolb et al., 2025; Rezaei et al., 2025). For iGABASnFr2 confocal imaging, slices were placed in a submersion chamber (Warner Instruments) of a custom Prior Open-scope microscope equipped with an X-light V2 Spinning disk confocal (Crest-optic, 89North LDI). Responses were imaged onto a Prime95B (Photometrics) camera with the same stimulus protocol as described for epifluorescence imaging. Responses were imaged using a 60× water-immersion objective (Olympus) with a 2× magnifier at a 100 Hz frame rate.

### IPSC recordings

IPSCs were recorded similar to previous studies (Ryner et al., 2023). Briefly, whole-cell patch-clamp recordings were made using the same system as live-slice imaging, with aCSF containing the AMPA receptor antagonist DNQX and the NMDA receptor antagonist APV. Pyramidal neurons were identified by morphology in layer V of the cortex, and whole-cell patch-clamp recordings using 2–5 MΩ borosilicate glass electrodes were established using an internal solution containing the following (in mM): 140 CsMs, 10 HEPES, 5 NaCl, 0.2 EGTA, 5 QX314, 1.8 MgATP, and 0.3 NaGTP, pH 7.25 (osmolarity 290 mOsm/L). In voltage-clamp mode, neurons were held at 0 mV (the reversal potential for glutamate receptors) to isolate IPSCs, and then IPSCs were evoked by electrical stimulation identical to the live-cell imaging above.

### Deconvolution

Averaged, normalized 10 stimuli 100 Hz iGABASnFr1 and iGABASnFr2 traces were used to deconvolve out the sensor properties from the underlying GABA kinetics. We assume that the recorded fluorescence signal is a convolution of the biological time-course of GABA in the extracellular space and the iGABASnFr response function, being a function of the *K*_on_ and *K*_off_ rates as follows:

$$F_{\mathrm{Fluorescence}}\;=\;I_{\mathrm{GABASnFr}\_\mathrm{response}}(K_{\mathrm{on}},K_{\mathrm{off}})\;*\;\mathrm{GABA}.$$

We assume the underlying GABA kinetics are the same, independent of which sensor is expressed. Based on published data, we assume the affinity of the sensors are iGABASnFr1 *K*_d_ = 30 µM (Marvin et al., 2019) and iGABASnFr2 *K*_d_ = 6.4 µM (Kolb et al., 2025). Using the relation that *K*_d_ is the balance of *K*_off_/*K*_on_ and the affinity relationship between iGABASnFr1 and iGABASnFr2, we can reduce the variables to 3: *K*_on_iGABASnFr1_, *K*_on_iGABASnFr2_, and the Response function amplitude *A*. Normalized responses are used as the sensors have different maximum Δ*F*/*F* responses (Kolb et al., 2025), making the fluorescence values not comparable. To enable the comparison between the sensors, the response function amplitude variable *A* is used as a scaling factor between the responses. Using the MATLAB (MathWorks) *fmincon* optimization function to minimize the difference between the iGABASnFR1 GABA response and iGABASnFR2 GABA response results in the iGABASnFr response functions. The response functions were then validated on a second dataset, which showed matching deconvolved GABA responses from iGABASnFr1 and iGABASnFr2 recordings, and applied to 1, 5, and 10 stimuli at 100 Hz traces (Fig. 2).

### Modeling

We developed a GABA diffusion model that was minimally adapted from a previous model of glutamate diffusion (Diamond, 2005; Thomas et al., 2011; Armbruster et al., 2020) written in MATLAB. Briefly, the model simulates a single synapse and a single-stimulus GABA release event, releasing 5,000 independent GABA molecules that originate from a point source in a 320 nm diameter synaptic cleft. For each 1 µs time step, each GABA molecule diffuses randomly, with a diffusion coefficient of 0.253 µs^2^/ms. For the synaptic cleft, diffusion is restricted to two dimensions but unrestricted once the molecule leaves the synaptic cleft. Outside the synaptic cleft, diffusion was scaled by the extracellular volume fraction (0.21) and the extracellular tortuosity (*λ* = 1.55). All of these parameters were unchanged from the existing, established model. Compared with the previous glutamate diffusion models, GAT density (∼1 µM) was reduced 100-fold compared with glutamate transporters (100 µM). This is based upon estimates of 800–1,300 per µm^2^ GAT-1 molecules at boutons, with additional reduction due to intracellular localization of 40% of GATs and reduced expression in other cell types (Chiu et al., 2002; Melone et al., 2015). For simplicity, we assume a homogenous distribution of GAT transporters in the extracellular space. GAT affinity and transport kinetics are very similar to GLT-1 [4–20 μM GAT1 (Radian and Kanner, 1983; Mager et al., 1993), 12 μM GLT-1 (Bergles and Jahr, 1997; Bergles et al., 2002), and 70–160 ms GAT1 (Mager et al., 1993) vs 20–70 ms GLT-1 (Wadiche et al., 1995; Bergles et al., 2002), respectively] and were left unchanged from the existing model.

### Analysis

Data were analyzed using MATLAB (MathWorks) and Origin (Originlab). For low magnification (10×) imaging data, the central quadrant (25−75%) of the camera field of view was isolated, spatially averaged downsampled 10-fold to reduce data size, and averaged across sweeps. Δ*F*/*F* traces were created using the 1, 5, and 10 stimuli traces divided by the no stimulus trace. Due to different bleaching kinetics, the first sweep was excluded. For epifluorescence traces an additional mono-exponential bleaching correction was applied (as needed) based on the prestimulus frames and frames at the end of the 3 s acquisition.

iGABASnFr decay times were measured using the *T*_80_–*T*_20_ time from the end of the stimulus. Traces were normalized 0–1, from the end of the stimulus. Traces were linearly extrapolated 10-fold, and 80 and 20% times were found. For analysis of peak versus decay, downsampled images were used to generate the average Δ*F*/*F* images. For each pixel, the signal-to-noise ratio (SNR) was calculated (peak Δ*F*/*F* divided by standard deviation of prestimulus frames). Regions of interest (ROIs) were defined by binning the SNR [bin size of 2 (minimum SNR of 2)]. Ten stimuli at 100 Hz ranged from SNR of 2 to 36. For each bin, an average trace was created with peak and decay quantified. Minimal response kinetics were calculated from single stimuli in minimal SNR bin datasets.

For confocal data, the individual sweeps were aligned with NoRMCorre algorithm (Pnevmatikakis and Giovannucci, 2017). Following averaging, stimulation responses were divided by the no stimulus traces for bleaching correction after subtracting the dark noise and autofluorescence to create Δ*F*/*F*_0_ datasets. To establish ROIs from confocal iGABASnFr2 imaging, aligned fluorescence images were averaged and smoothed with a 1.5 pixel 2D Gaussian spatial filter and a one-sided Gaussian temporal filter (sigma, 50 ms). Next the images were divided by a 15 pixel radius spatial filtered version of the data (pixel size, 92 nm) to highlight responsive areas over the background. The two frames poststimulation were averaged, and all responses >4 standard deviations over the baseline, >6 pixels, and a circularity of >0.75 were defined as sites of GABA release. ROI masks were then applied to the 1, 5, and 10 stimuli at 100 Hz Δ*F*/*F* traces.

### Statistics and reproducibility

Individual statistical tests are noted throughout the manuscript and figure legends. Linear mixed models were used to account for potential nonindependence of slices coming from the same mouse. We used the *lmer* function in R to fit linear mixed models. For all models, statistical significance was defined as *α* < 0.05. When significant main effects or interactions were found, paired or two-sample *t* tests or two-way repeated-measures ANOVA with Tukey's posttest were made to determine differences between specific groups as needed. Sample sizes (slices/cells) are listed for each experiment in the figure labels or text; all experiments are from ≥3 mice, with exact *n* values for mice, slices, and imaging fields of view noted throughout in the figure legends. Error bars indicate standard error of the mean (SEM).

## Results

### Measuring extracellular GABA dynamics in the cerebral cortex using iGABASnFr imaging

Many studies have attempted to use electrophysiological recordings of GABAR activity (Jensen et al., 2003; Chiu et al., 2005; Kinney, 2005), GAT transporter currents (Kinney and Spain, 2002), sniffer patches (Sylantyev et al., 2020; Magloire et al., 2023), and other indirect approaches to infer extracellular GABA dynamics (Isaacson et al., 1993; Scanziani, 2000; Quick, 2002; Overstreet and Westbrook, 2003; Beenhakker and Huguenard, 2010). Across these techniques, there are significant differences in observed GABA dynamics, ranging across multiple orders of magnitude. Recent advances in iGABASnFr imaging have now enabled direct visualization of GABA dynamics in the extracellular space, providing a rapid, optical approach to quantify extracellular GABA. This has been used to report GABA dynamics in vivo (Shimoda et al., 2024; Rezaei et al., 2025), pre-ictal GABA transients (Magloire et al., 2023), and circadian GABA rhythms (Patton et al., 2023). Both Magloire et al. and Shimoda et al. reported that GABA activity persisted for an extended time following interictal spikes, far greater than iGluSnFr or electrical signals. These findings suggested that extracellular GABA may be slower and more variable than had been previously appreciated. Magloire et al. also validated their iGABASnFr findings with sniffer-pipette recordings, validating using iGABASnFr to report extracellular GABA dynamics.

To better understand the kinetics of extracellular GABA dynamics in the cortex, we expressed the neuronally targeted GABA sensor, iGABASnFR1, in layer IV/V of the somatosensory cortex in 4–6-week-old mice via intracortical AAV injection (AAV1-hsyn-iGABASnFR1; Fig. 1*A*). Two to four weeks following injection, acute brain slices were prepared for iGABASnFR imaging. GABA release was induced via local electrical stimulation (2× threshold stimulation) using a bipolar stimulating electrode placed in layer V in the presence of AMPA and NMDA receptor antagonists (DNQX 20 μM and APV 50 μM). Spatially averaged iGABASnFr1 ΔF/F responses were imaged (10× magnification) and showed a stimulation number-dependent increase in iGABASnFr1 response [Fig. 1*B*; 1, 5, and 10 stimuli delivered at 100 Hz; 0.15 ± 1.2 × 10^−4^, 0.44 ± 3.2 × 10^−4^, 0.65 ± 4.1 × 10^−4^ Δ*F*/*F* (%), respectively; *N* = 21 slices, 7 mice] demonstrating the ability to quantify extracellular GABA across a broad dynamic range (Fig. 1*C*). This epifluorescent assay of iGABASnFr measures the spatially averaged (0.6 mm^2^ area) response of many activated synapses, representing the average extracellular GABA signal across the neuropil. iGABASnFr1 Δ*F*/*F* signals had prolonged decay times (quantified by the *T*_80_–*T*_20_ decay time) with increasing numbers of stimuli (Fig. 1*B*, inset, *D*; 299 ± 33 ms, 628 ± 61 ms, 854 ± 72 ms for 1, 5, and 10 stimuli at 100 Hz, respectively; *N* = 20 slices, 7 mice). This suggests that, once released, GABA persists in the extracellular space for a prolonged period following brief bursts of activity. To ensure that GABA signal duration was not due to sustained release of GABA from prolonged inhibitory interneuron activity, we made recordings from parvalbumin (PV) interneurons during identical electrical stimulation used to quantify iGABASnFr. We found brief inhibitory synaptic currents, but not action potentials, in PV interneurons following stimulation. In addition, we quantified the frequency of sIPSCs before and after burst of activity and found no significant differences (Fig. S2). This suggests that prolonged iGABASnFr signals are not due to ongoing GABA release but rather to the slow clearance of GABA from the extracellular space. It is important to note that the use of optical tools to image neurotransmitters can both alter neurotransmitter dynamics via buffering (Armbruster et al., 2020) and provides a view of neurotransmission filtered by the properties of the sensor itself.

**Figure 1.**
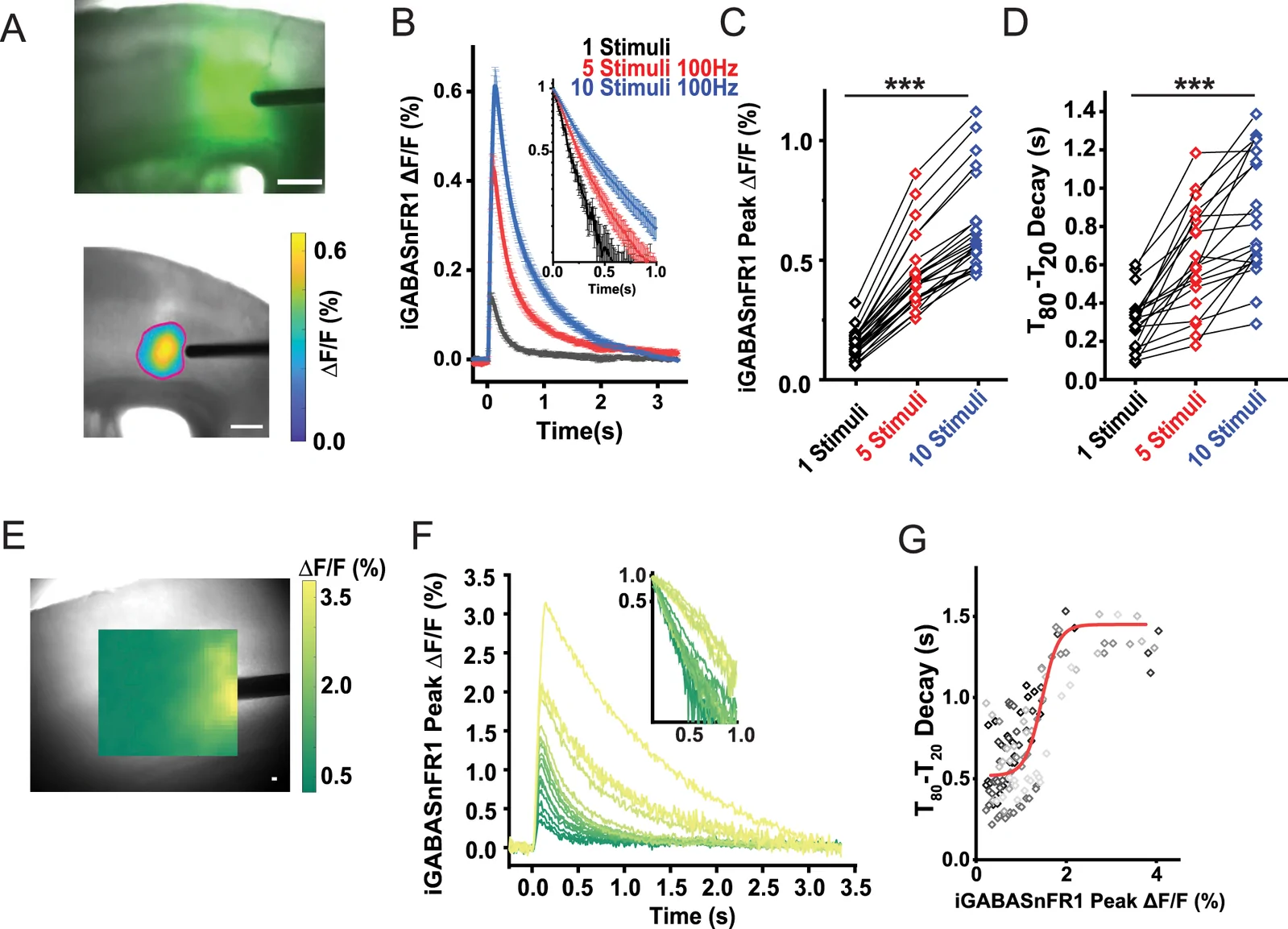
iGABASnFr decay is slow and modulated by local GABA load. ***A***, 4× bright-field image and basal fluorescence image of iGABASnFR1 showing the area of expression. Scale bar, 125 μm. Below a second 4× representative bright-field image with pseudocolored Δ*F*/*F* iGABASnFR1 response to 10 stimuli at 100 Hz. Scale bar, 125 μm. ***B***, Average iGABASnFR1 Δ*F*/*F* traces in response to 1, 5, and 10 stimuli at 100 Hz with a semi log inset of the normalized fluorescent decay trace. ***C***, iGABASnFR1 peak Δ*F*/*F* (%) increases with stimuli number. LMM, stimuli dependence *p* < 2 × 10^−16^. ***D***, *T*_80_–*T*_20_ decay of iGABASnFr1 traces shows stimulus dependence LMM, stimuli dependence *p* < 7.9 × 10^−13^. *N* = 7 mice; 21 slices. ***E***, Bright-field image with iGABASnFR1 response binned by their SNR and pseudocolored for 10 stimuli at 100 Hz. ***F***, Representative example of a single slice's DF/F response for each ROI on the matching color scale. ***G***, Average ROI iGABASnFr1 peak and decay in response to 10 stimuli at 100 Hz for 10 slices fitted with a sigmoidal curve, individual slices color coded. *N* = 6 mice; 9 slices.

### GABA persists longer in the extracellular space in areas of heightened release

iGABASnFr imaging enabled us to quantify the persistence of GABA in the extracellular space and how this is shaped by the number of stimuli and the local GABA concentration. Published recordings suggest IPSC kinetics are slowed by increased density of activated release sites. This has been shown both by recruiting more axons via increased stimulus intensity and by the difference between evoked IPSCs and spontaneous/mini IPSCs (Overstreet and Westbrook, 2003). To determine whether the iGABASnFr decay times were associated with increased amounts of GABA release rather than simply the number of stimuli delivered, we correlated local iGABASnFR1 peak Δ*F*/*F* with *T*_80_–*T*_−20_ decay times. ROIs were defined for the iGABASnFr1 Δ*F*/*F* responses to 10 stimuli delivered at 100 Hz based on their peak SNR (peak response divided by standard deviation of the prestimulus baseline). For each ROI, the peak Δ*F*/*F* and decay times were quantified (Fig. 1*E*–*G*). The iGABASnFR peak Δ*F*/*F* showed a sigmoidal relationship with decay time [minimum, 526 ± 33 ms; maximum, 1,354 ± 54 ms; half max Δ*F*/*F* (%), 1.5 ± 0.063; slope, 0.21 ± 0.057; *R*^2^ = 0.669; Fig. 1*G*], suggesting that the local extracellular GABA level influences how long GABA persists in the extracellular space. The slowed decay time is similar to observed time constants for dye diffusion in the cortex (Binder et al., 2004), suggesting overwhelmed GAT transport capacity and diffusion-mediated clearance.

### Deconvolution of epifluorescence iGABASnFr imaging reflects the underlying GABA transients

It is critical to determine whether the observed slow kinetics are due to the properties of iGABASnFr or reflect the underlying GABA dynamics. Slow sensor kinetics can aberrantly show summation due to sensor properties rather than actual GABA accumulation (Helassa et al., 2018). However, summation due to slow sensor kinetics should not cause a stimulus-dependent change in the decay kinetics; this has been seen by differences in iGluSnFr kinetics across brain regions (Pinky et al., 2018). To test this idea computationally, we used recorded responses for two sensors, high-affinity-SF-iGluSnFr [decay dominated by sensor properties, trace from Armbruster (2026)] and iGABASnFr1 (Fig. 1). A 10-stimulus, 100 Hz train of high-affinity-iGluSnFr can be largely represented by a weighted linear summation of a single-stimulus response, suggesting that there is not a universal distortion in sensors kinetics to repeated stimuli. However, the iGABASnFr1 trace shows a large divergence between the summation of a single-stimulus response and the 10-stimulus trace, likely due to changes in GABA clearance rather than sensor properties (Fig. S3).

While iGABASnFr fluorescence responses are very slow in areas of elevated GABA release (Fig. 1*D*, 854 ± 72 ms), single-stimulus responses in areas of sparse GABA release (identified by iGABASnFr SNR of 2–4 standard deviations over the baseline in response to one stimulus, corresponding to 36 ± 4% of the maximum response within each slice.) show a *T*_80–20_ decay kinetic of 188 ± 15 ms (*N* = 6 slices). This waveform is still a convolution of the underlying GABA clearance dynamics and the properties of the sensor. While it does not inform the underlying GABA kinetics, it does provide an upper bound on the kinetics of the sensor. This would suggest the slow decays, which are multiples of this kinetic, are mostly due to GABA clearance rather than properties of the sensor. iGABASnFr2, a higher affinity sensor (30 vs 6 μM affinity for iGABASnFr1 and iGABASnFr2, respectively; Kolb et al., 2025), 30 versus 6 μM with enhanced temporal properties comparable to the original iGluSnFr (Helassa et al., 2018), enables us to compare the kinetics using two different sensors. To characterize the underlying GABA transients, rather than those of the sensor, we used a mathematical deconvolution approach to isolate the GABA transient from the kinetics of the sensors. By using two different affinity sensors (Fig. 2*A*) and assuming that their affinities are a product of their kinetics (*K*_on_/*K*_off_; Helassa et al., 2018), we deconvolved the filtering effect of the sensors and estimate the underlying GABA kinetics (Fig. 2*B*). This approach estimates the decay time constant for the sensors at 37 and 66 ms for iGABASnFr1 and iGABASnFr2, respectively. For 10 stimuli delivered at 100 Hz, the deconvolved GABA signals had similar decays to the raw iGABASnFr signal (Fits on average traces, T_80-20_ iGABASnFr1, 1,104 ms vs iGABASnFr1_deconvolved 1,196 ms and iGABASnFr2, 978 ms vs iGABASnFr2_deconvolved 1,084 ms) but revealed multiple components in the iGABASnFr decay. For smaller stimuli with faster kinetics, a greater fraction of the kinetics is due to the sensor properties. This is seen by applying the deconvolution function to the one and five stimuli at 100 Hz which revealed a fast component of decay which diminishes with increasing stimuli (Fig. 2*C*). Because these kinetics are closer to the kinetics of the sensor, they are more strongly affected by the sensor properties.

**Figure 2.**
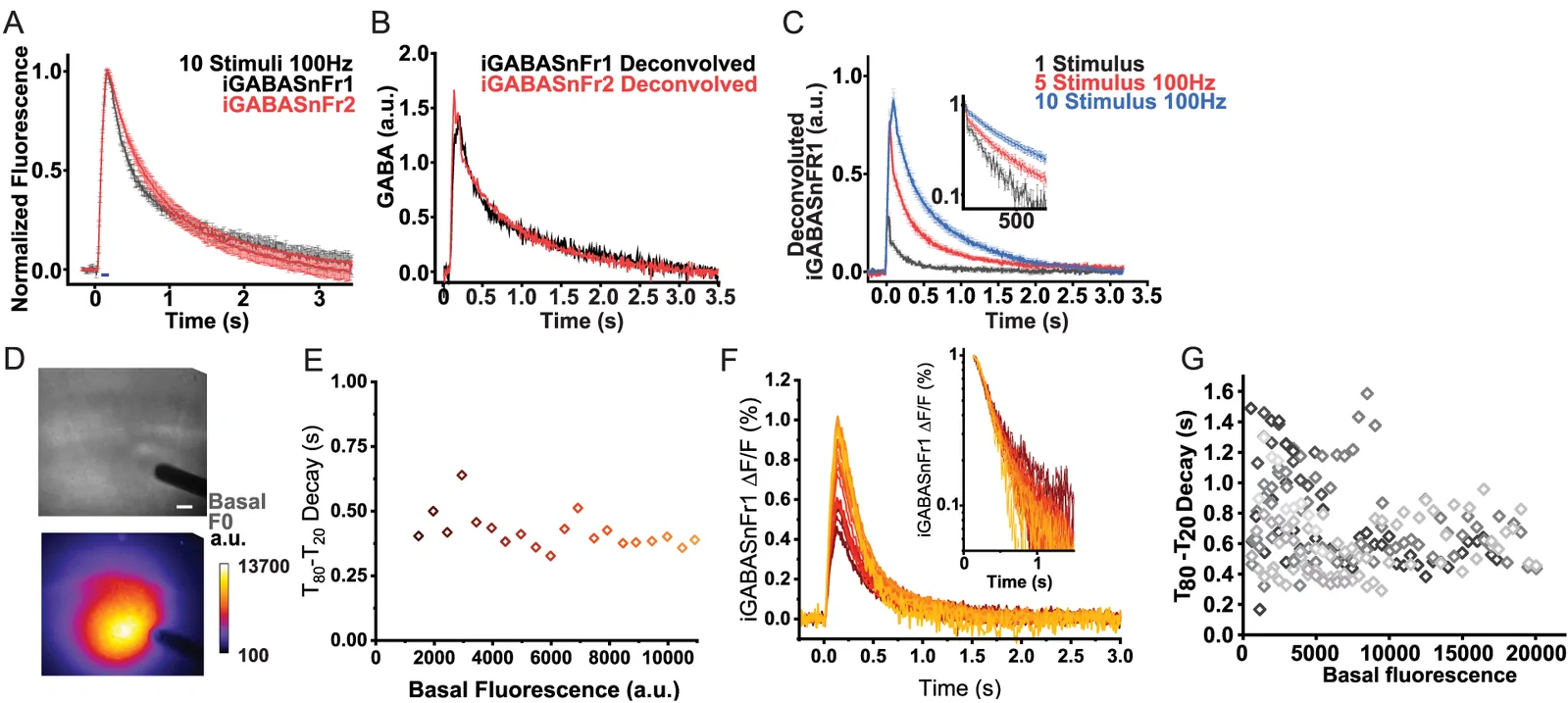
iGABASnFr fluorescence reflects underlying GABA. ***A***, Normalized, averaged iGABASnFr1 and iGABASnFr2 traces in response to 10 stimuli at 100 Hz show slight differences in their kinetics due to the differing sensor affinities. Using the relative affinities of the two sensors along with the normalized traces, we derived the following. ***B***, Average deconvolved traces for 10 stimuli at 100 Hz for iGABASnFr1 and iGABASnFr2 show similar kinetics for both sensors. ***C***, Using the deconvolution function established in ***B***, we deconvolved the average 1-, 5-, and 10-stimulus, 100 Hz iGABASnFR1 traces from Figure 1*B*. ***D***, In order to rule out iGABASnFr expression levels driving the decay kinetics, we compared the basal fluorescence (top) and the Δ*F*/*F* response to 10 stimuli at 100 Hz (bottom). Scale bar, 2.6 μm. ***E***, Binned *T*_80_–*T*_20_ Decay (ms) vs basal fluorescence in response to 10 stimuli at 100 Hz for an example slice with traces in ***F***, in matching colors shows little change in decay with changes in basal fluorescence. ***G***, *T*_80_–*T*_20_ Decay (ms) vs basal fluorescence in response to 10 stimuli at 100 Hz for 10 slices shows no correlation of the decay and the basal fluorescence *N* = 6 mice; 10 slices.

Additionally, we have previously shown that neurotransmitter sensors expressed at high levels can artificially prolong extracellular dynamics via buffering by the sensor (Armbruster et al., 2020). Because extracellular GABA dynamics reported by iGABASnFR show very long decay times, we considered the possibility that this was due to high sensor expression levels. To test this, we took the baseline iGABASnFR fluorescence prior to stimulation, a proxy for sensor expression, and asked if it was correlated to the decay time (Fig. 2*D*). For both individual (Fig. 2*E*,*F*) and pooled slices (Fig. 2*G*), the baseline fluorescence showed no correlation with the decay (*R*^2^ = 0.002). This suggests that, within our experimental regime, the decay kinetics that we observe are primarily driven by the underlying GABA dynamics and are not due to excessive iGABASnFR expression distorting the response (Fig. 2*F,G*). iGABASnFr peak shows a much clearer relationship with iGABASnFr1 decay times than the basal fluorescence. These important controls show that the slow epifluorescent iGABASnFr kinetics following 10 stimuli trains are a suitable proxy for quantifying spatially averaged GABA dynamics in the extracellular space. For smaller stimuli with faster dynamics, the raw epifluorescent iGABASnFr traces have a larger component driven by the sensor properties and in these cases the iGABASnFr traces overestimate the underlying GABA dynamics.

### Phasic inhibition does not reflect extracellular GABA summation

IPSC recordings show significant depression with repeated stimuli (Varela et al., 1999; Fig. 3*A*), which has previously been interpreted as suppression of GABA release (Varela et al., 1999). iGABASnFr1 imaging, on the other hand, suggests continued release of GABA during bursts of activity that lead to extracellular GABA accumulation (Fig. 1*B*). Therefore, we asked how neuronal GABA IPSCs summate or depress during bursts of activity and how that relates to extracellular GABA levels during equivalent stimuli (Fig. 3*B*). We made whole-cell patch-clamp recordings from layer V pyramidal neurons and isolated IPSCs by holding the cell at the glutamate receptor reversal potential (0 mV) while also pharmacologically blocking AMPA (20 µM DNQX) and NMDA receptors (50 µM APV). As with iGABASnFR1 imaging, GABA release was induced via local stimulation with a bipolar electrode (Fig. 3*A*). iGABASnFr1 imaging showed that the integrated signal showed significantly more summation from 1 to 5 and 10 stimuli, as compared with integrated IPSC currents (iGABASnFr1 area under the curve: 5.0 ± 0.7 and 8.6 ± 1.3 fold increase of over 1 stimuli for 5 and 10 stimuli, respectively, *N* = 21 slices from seven mice; IPSC integrated charge: 2.5 ± 0.32 and 3.5 ± 0.6 fold increase over 1 stimuli for 5 and 10 stimuli, *N* = 13 cells from four mice; LMM *p* = 0.0002; Fig. 3*C*). When we examined the peak iGABASnFR1 Δ*F*/*F* and peak amplitude of the IPSC, we found that iGABASnFr peaks summate (iGABASnFr1 peaks normalized to 1 stimuli, 3.2 ± 0.1, 4.6 ± 0.3 for 5 and 10 stimuli) while IPSC peaks depress (normalized to 1 stimulus 0.55 ± 0.25, 0.38 ± 0.22 for 5 and 10 stimuli LMM *p* < 2 × 10^−16^, iGABASnFr *N* = 21 slices from seven mice, IPSC *N* = 13 cells from four mice; Fig. 3*D*). This suggests that even though GABA release and extracellular accumulation increase during repeated stimulation, this is not reflected by corresponding increases in phasic inhibitory synaptic transmission. Additionally, this suggests our stimulation protocol may be biased toward activating PV interneurons, which drive synaptic inhibition that depresses with repeated stimuli (Ma et al., 2012).

**Figure 3.**
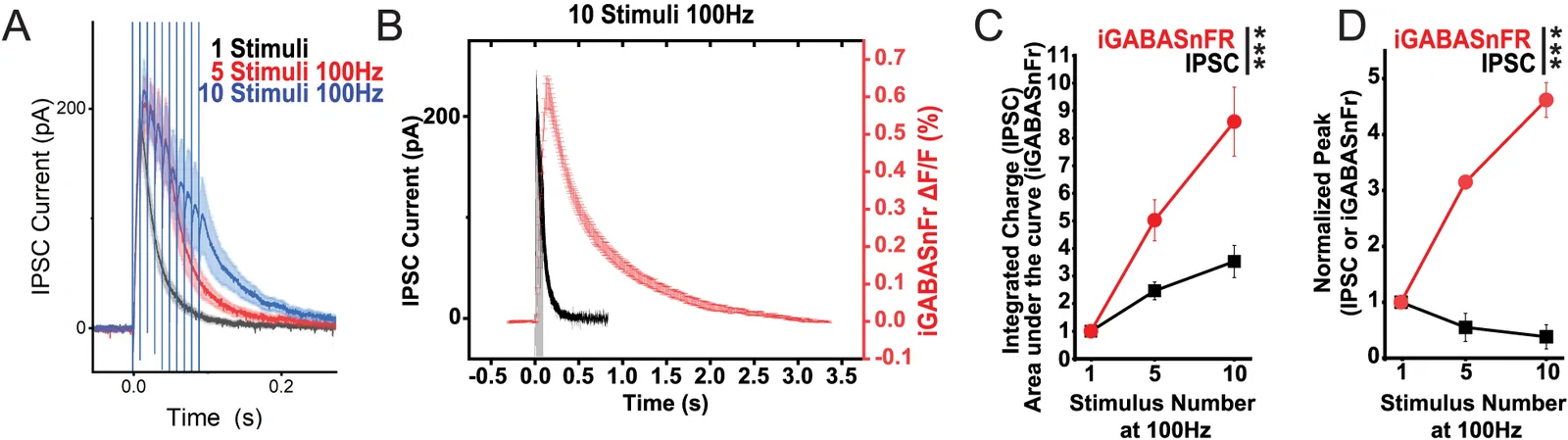
iGABASnFr revels a disconnect between GABA summation and IPSCs. ***A***, Stimulus-evoked IPSCs recorded from Layer V pyramidal neurons, for 1, 5, and 10 stimuli at 100 Hz shows much faster decays and reduced summation across stimuli. ***B***, Overlay of IPSC kinetics compared with iGABASFnR1 for 10 stimuli at 100 Hz shows differential kinetics of the two readouts. ***C***, IPSC integrated charge plotted against iGABASnFr1 area under the curve shows significantly greater summation of stimuli for iGABASnFr1 compared with IPSCs (LMM *p* < 2.7 × 10^−4^). ***D***, IPSC peaks show depression with stimuli number compared with summation for iGABASnFr1. LMM *p* < 2 × 10^−16^ *N* = 13 cells from 4 mice, *N* = 21 slices from 7 mice. *p < 0.05, **p < 0.01, ***p < 0.001.

### GATs play a small but significant role in removing extracellular GABA in the cortex

GAT1 and GAT3 are both expressed in the somatosensory cortex (Minelli et al., 1995; Scimemi, 2014), with GAT1 primarily expressed in presynaptic neurons and GAT3 primarily expressed in astrocytes (Minelli et al., 1996; Conti et al., 2004; Fattorini et al., 2020; Bhatt et al., 2023). Loss of either transporter induces changes in network activity and pathologies, including epilepsy and ataxia (Goodspeed et al., 2020; Park et al., 2024). To determine the contribution of each transporter to extracellular GABA dynamics, we sequentially inhibited GAT1 [25 µM NO-711 (Matthews et al., 2009)], GAT3 [50 µM SNAP-5114 (Moldavan et al., 2017; Zafar and Jabeen, 2018)], and both together while quantifying iGABASnFR1 peaks and decays with epifluorescence imaging in response to 10 stimuli delivered at 100 Hz (Fig. 4*A*–*D*). Inhibiting GAT1 with NO-711 significantly slowed iGABASnFR1 *T*_80_–*T*_20_ decay time (0.55 ± 0.07 vs 0.72 ± 0.08 s, *N* = 6 slices; paired-sample *t* test, *p* = 0.0165; Fig. 1*A*). Inhibiting GAT3 with SNAP-5114 showed no significant effect on the decay time (0.78 ± 0.1 vs 0.9 ± 0.15 s *N* = 5 slices; paired-sample *t* test, *p* = 0.39; Fig. 4*B*). Inhibiting both GAT1 and GAT3 showed a significantly bigger effect, normalized to control, than inhibiting GAT1 alone (Fig. 4*C*,*D*; ratio of decay to control, NO711 1.35 ± 0.13, SNAP5114 1.14 ± 0.15, NO711 + SNAP5114 1.80 ± 0.20, one-way ANOVA Tukey test, NO711 vs NO711 + SNAP5114 *p* < 6.8e-4, SNAP5114 vs NO711 + SNAP5114 *p* = 0.002, NO711 vs SNAP514 *p* = 0.63, *N* = 10 slices). This suggests the smaller role of GAT3 compared with GAT1, likely in proportion to their comparative expression levels in the cortex (Durkin et al., 1995). A single stimulation showed no effect of inhibiting both GAT1 and GAT3 (Fig. 4*E*, paired *t* test control vs NO711 + SNAP5114, *p* = 0.15371), but 5 stimuli at 100 Hz showed similar results to the 10 stimuli 100 Hz (Fig. 4*F*, paired *t* test control vs NO711 + SNAP5114, *p* = 0.00497) and 5 stimuli at 100 Hz (Fig. 4*F*,*G*). GAT inhibition decreased iGABASnFR1 peak Δ*F*/*F* (Fig. S4). By comparison, when glutamate transporters are inhibited with the EAAT inhibitor 1 µM TFB-TBOA, iGluSnFr shows a large increase in fluorescence (Fig. S5) due to fast glutamate uptake during the stimulus train. To test if changes in peak iGABASnFR Δ*F*/*F* are due to photobleaching or decreases in GABA release, we plotted the average iGABASnFR peak for each sweep, for each condition (control, NO-711, NO711 + SNAP5114; Fig. S4). We observed a linear decrease in peak Δ*F*/*F* fluorescence per sweep that is independent on GAT inhibition. This suggests the changes are largely due to photobleaching rather than changes in release. Together, this shows that GATs play a small but significant role in removing GABA from the extracellular space, with little to no effect on peak GABA levels.

**Figure 4.**
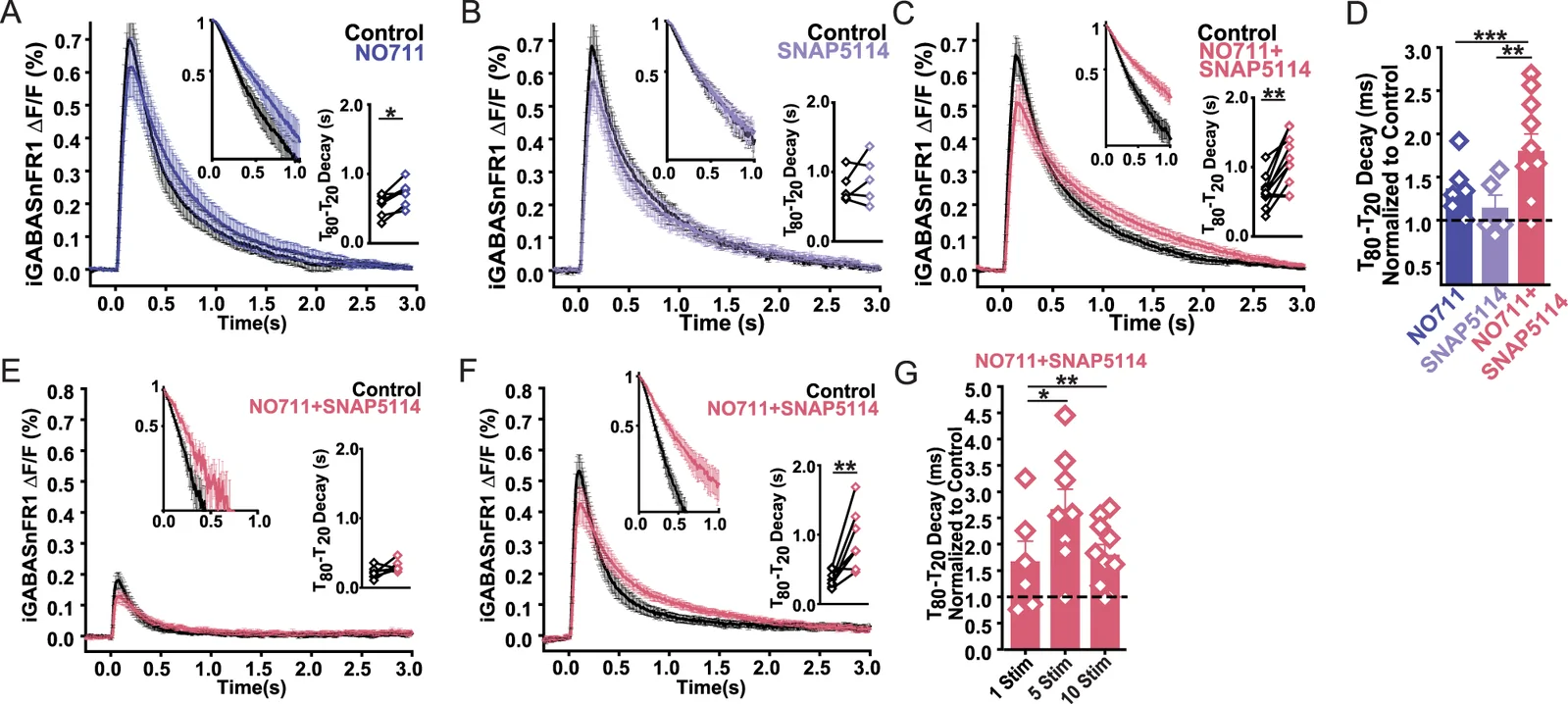
iGABASnFr imaging reveals the small role of GABA transport to extracellular GABA clearance. ***A***, Average traces of iGABASnFR1 in response to 10 stimuli at 100 Hz while inhibiting GAT1 with in 25 μM NO711 (***B***), GAT3 with 50 μM SNAP5114, and (***C***) both GAT1 and GAT3, with a semi log inset of the normalized fluorescent decay trace and a line series inset of the T_80_–T_20_ Decay (s). Paired *t* test (*T*_80_–*T*_20_ Decay) NO711, *p* < 0.017, *N* = 3 mice, six slices, SNAP5114 *p* = 3 mice, five slices, NO711 + SNAP5114, *p* < 0.002, *N* = 10 slices, six mice. ***D***, iGABASnFr decay times for each slice normalized to control shows the additive effect of GAT1 + GAT3 inhibition, one-way ANOVA Tukey test NO711 versus NO711 + SNAP5114, *p* < 6.8e^−4^, SNAP5114 versus NO711 + SNAP5114, *p* = 0.002. ***E***, Traces of iGABASnFR Δ*F*/*F* (%) in response to one stimulus delivered at 100 Hz in in both control and in GAT1 + 3 inhibitor group. Paired *t* test [*T*_80_–*T*_20_ Decay (s)] control versus NO711 + SNAP5114, *p* = 0.15371. ***F***, In response to five stimuli delivered at 100 Hz paired *t* test [*T*_80_–*T*_20_ Decay (s)] control versus NO711 + SNAP5114, *p* = 0.00497. ***G***, *T*_80_–*T*_20_ Decay (s) normalized to control. LMM variable Stim number **p ≤ 0.00364. Post hoc Tukey test [*T*_80_–*T*_20_ Decay (ms) Normalized to Control], 1 Stim versus 5 Stim *p* = 0.028, 1 Stim versus 10 Stim **p = 0.0095, and 5 Stim versus 10 Stim *p* = 0.80. *N* = 6 mice; nine slices. *p < 0.05, **p < 0.01.

### Modeling synaptic GABA clearance

Epifluorescence iGABASnFR1 imaging showed very slow extracellular GABA clearance with only small effects of GAT blockade, which is inconsistent with the effects of GAT1 inhibition on evoked IPSCs where it prolongs currents by 10 s of milliseconds (Rossi and Hamann, 1998; Calcagnotto et al., 2002; Overstreet and Westbrook, 2003; Keros and Hablitz, 2005). To better understand extracellular GABA dynamics and the role of GATs in GABA dynamics, we simulated GABA release, diffusion, and uptake using a computational model. We minimally adapted an established Monte Carlo simulation of extracellular glutamate dynamics (Armbruster et al., 2020) to model iGABASnFr, GATs, and GABA diffusion in the extracellular space for a single synapse in isolation. Based on experimental data suggesting much lower concentration of GATs compared with EAATs, we reduced the concentration of transporters in the model from 100 μM (glutamate; Lehre and Danbolt, 1998) to 1 μM (GABA; Chiu et al., 2002). Briefly, the model simulates the diffusion of 5,000 GABA molecules in the extracellular space, released from a single synapse. For each time step, each GABA molecule diffuses, and there is a chance for each molecule to bind, unbind the simulated sensor (iGABASnFr) or bind, unbind, and be transported by the simulated transporters (GATs). To visualize the model output, we defined three ROIs in the model, around the synapse (referred to as synaptic ROI, within 1 μm of the release site), the peri-synaptic (1–3 μm from the release site), and the full field (1,000 μm^3^; Fig. 5*A*). Simulated iGABASnFR2 responses in the synaptic ROI showed a rapid rise, increased peak, and faster decay compared with the full field, with the peri-synaptic ROI showing intermediate properties (Fig. 5*B*,*C*). The synaptic ROI traces agree with previous GABA models, showing rapid diffusion of GABA from the synaptic cleft (Quick, 2002), while the full field traces reflect the epifluorescence single-stimulus iGABASnFr responses (Fig. 1*B*). To test the role of GAT-mediated uptake in our simulation, we reduced GAT activity between 30 and 90% by reducing the number of transporters present. This significantly slowed the simulated iGABASnFR2 clearance for the full field but had no effect on GABA clearance within the synaptic ROI (Fig. 5*D*,*E*), suggesting that diffusion rather than transport dominates in the synaptic ROI. This is consistent with the lack of published data on the effects of GAT inhibition on mini IPSCs. Both mini IPSCs and our model address the kinetics of a single release site in isolation and thereby lack the summation of GABA from multiple release sites (evoked IPSCs, epifluorescence iGABASnFr) needed to robustly engage GATs. However, the cumulative transport of GATs in this model shows that GATs bind a fraction of the released GABA within the time-course of an IPSC (<30 ms; Fig. S6). This accounts for <10% of the total GABA clearance for a single release site, but if extracellular GABA summation occurs across multiple synapses, this could contribute to the effects of GAT inhibition on evoked IPSC. Together, our computational modeling suggests that GABA diffuses rapidly out from the synapse, where it can further diffuse or be transported by GATs.

**Figure 5.**
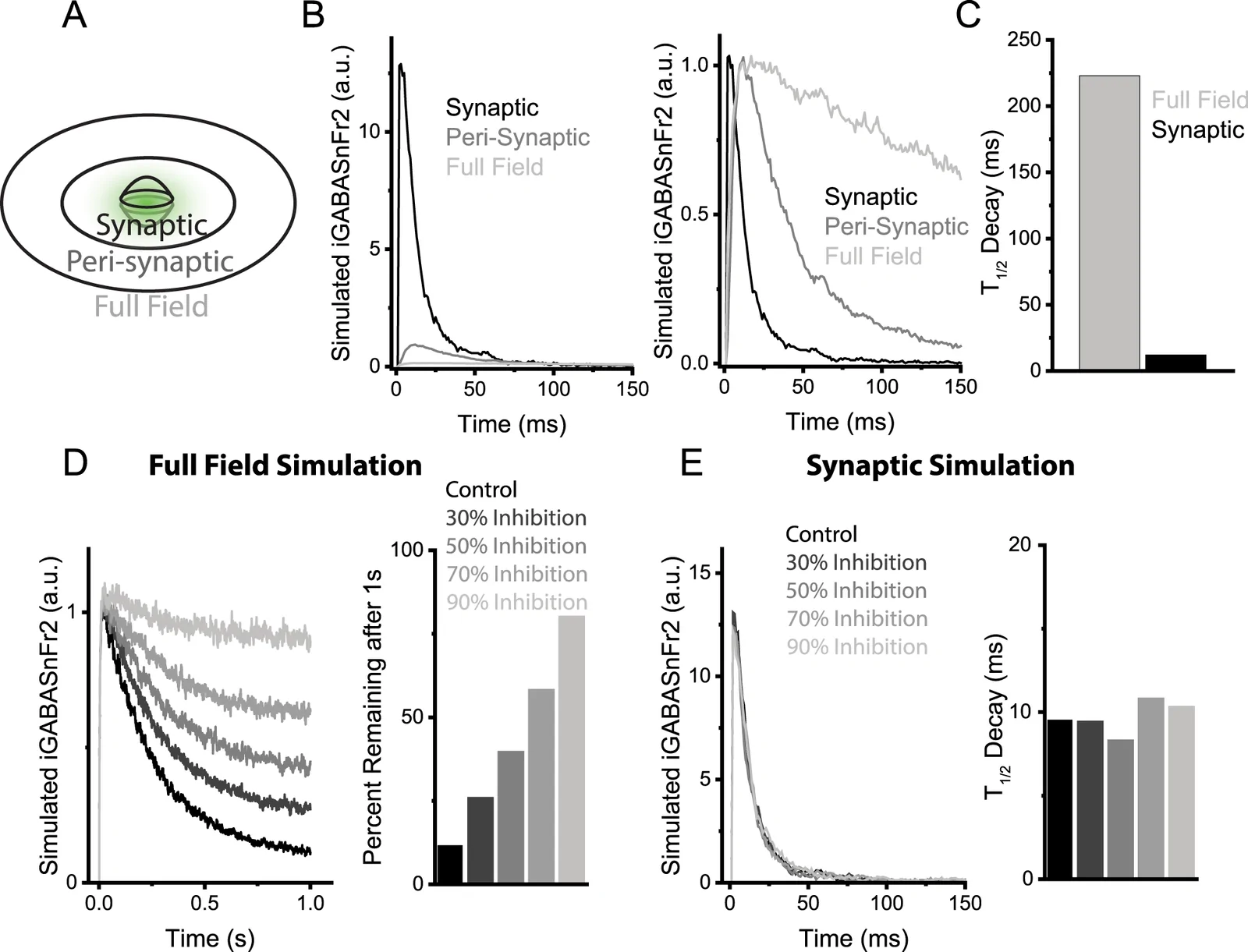
Monte Carlo modeling of synaptic GABA clearance. ***A***, Visualization of a Monte Carlo diffusion/clearance model of GABA in the extracellular space. GABA is released in a 320 nm synaptic cleft, and we have defined three ROIs (synaptic <1 µm, peri-synaptic 1–3 µm and full field). ***B***, Simulated iGABASnFr2 traces of synaptic, peri-synaptic, and full field ROIs both raw and normalized plots. The traces show faster iGABASnFr2 decays in the synaptic ROI compared with the full field, quantified in ***C***. ***D***, Model simulations with varying percentage inhibition of GATs (30–90%) to simulate GAT inhibitors show slowed clearance for full field ROIs, with greater percentage of simulated iGABASnFr2 signal remaining 1 s after stimulus. ***E***, Synaptic ROI shows no effect of GAT inhibition on the clearance kinetic.

### High-resolution iGABASnFr imaging confirms rapid GABA diffusion from release sites to the extrasynaptic space

To determine if modeling accurately predicts extracellular GABA dynamics, we used spinning disk confocal iGluSnFR2 (Kolb et al., 2025) imaging to visualize sites of evoked GABA release (Fig. 6*A*). iGABASnFR2 fluorescent responses were repeatedly imaged for a single stimulus, and putative sites of release were defined as small areas (>0.2 µm^2^ with >0.75 circularity) that rapidly increased Δ*F*/*F* following stimulation (within 1–2 frames, 10–20 ms), here termed Synaptic ROIs (Fig. 6*B*). Similar to the modeling results, iGABASnFR2 imaging showed a significantly larger peak around the sites of release (Synaptic ROI), as compared with the response seen across the entire field of view (Fig. 6*C*; peak Δ*F*/*F* 4.8 × 10^−4^, 2.2 × 10^−2^ for full field of view vs Synaptic ROI, LMM, *p* = 8.5 × 10^−16^, from 18 imaging fields from 11 slices from three mice). Additionally, iGABASnFR Δ*F*/*F* in the Synaptic ROI had significantly faster decay times as compared with the full field, again mirroring the computational findings (Fig. 6*D*; 259 ± 43 ms and 90 ± 12 ms for full field of view vs Synaptic, LMM, *p* = 0.0015 from 18 imaging fields from 11 slices from three mice). While the fluorescence decay of the Synaptic ROI is dominated by a fast decay, it also has a smaller slow phase likely resulting from the Synaptic ROI not being completely restricted to the release site due to optical resolution limits. This fast decay remains a convolution of the underlying GABA dynamic and the sensor properties, suggesting that iGABASnFr2 imaging can report comparatively rapid kinetics and suggesting that the slow decays seen in other experiments largely represent slow neurotransmitter dynamics and are influenced less by the sensor properties.

**Figure 6.**
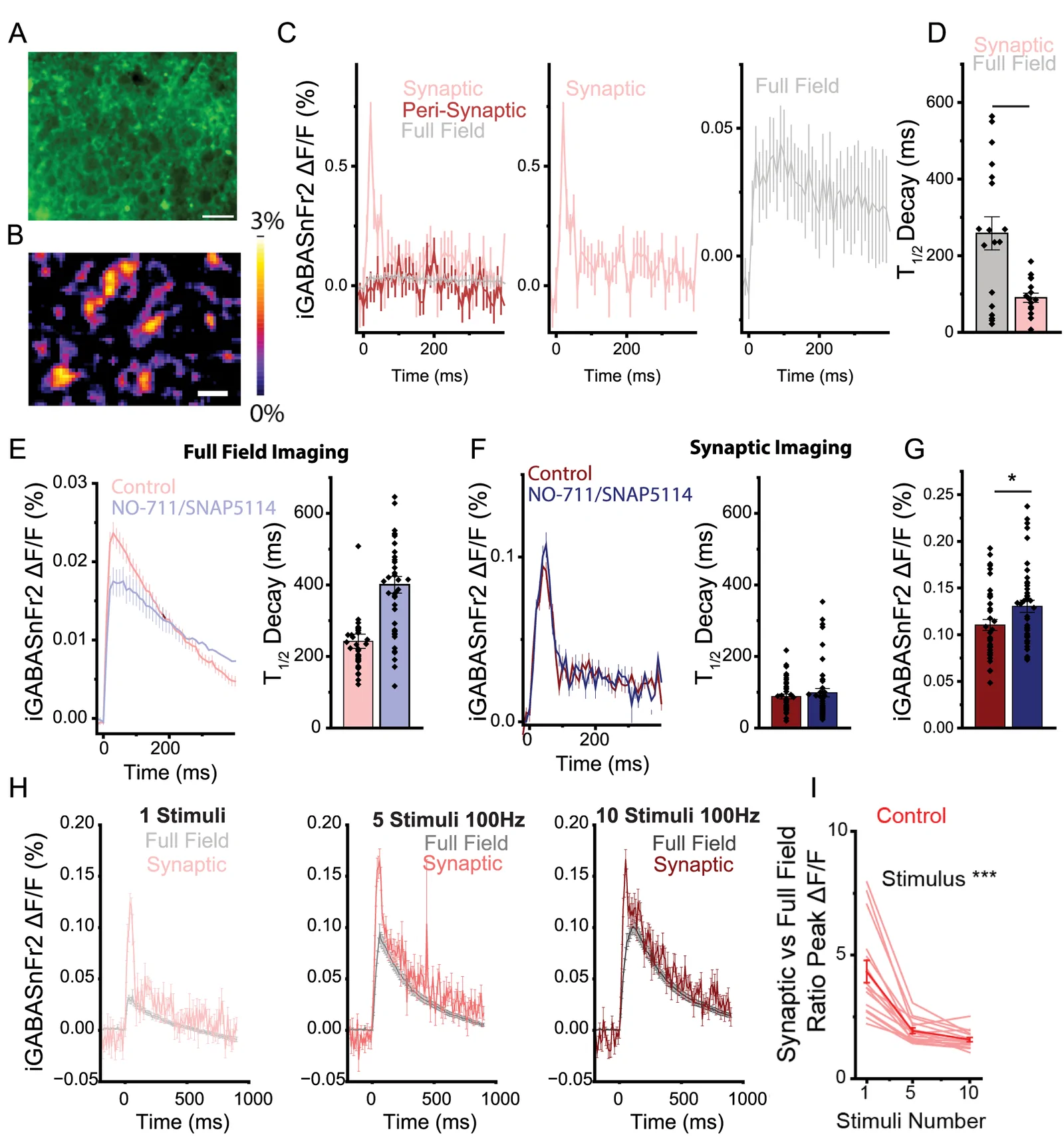
Confocal imaging of synaptic GABA clearance reveals the role of diffusion. ***A***, Spinning disk confocal imaging of iGABASnFr2 showing expression in the neuropil. Scale bar, 10 μm. ***B***, Cropped Δ*F*/*F* iGABASnFr2 image in response to a single stimulus shows responsive release sites. Scale bar, 1 μm. ***C***, Average synaptic, peri-synaptic, and full field ROIs confocal iGABASnFr2 traces show faster iGABASnFr2 decays quantified in ***D***. LMM *p* = 0.0015, *N* = 18 imaging fields from 11 slices from four mice. ***D***, Confocal imaging of iGABASnFr2 with or without NO711 and SNAP5114 shows slowed decay for the Full Field ROI *p* = 5E-07. ***E***, The same imaging for Synaptic ROIs shows no effect on the decay with NO711/SNAP5114 inhibition. *p* = 0.29. *N* = 43, 44 imaging field, from 14 slices from four mice. ***F***, Synaptic ROIs reveal a small change in peak iGABASnFr2 signal, representing fast GABA clearance at synapses. LMM *p* = 0.0263 *N* = 43, 44 imaging fields from 14 slices, three mice. ***G***, Synaptic compared with full field average iGABASnFr2 traces, comparing the enrichment of iGABASnFr2 signal at synapses. ***H***, Increasing stimuli number reduce the difference between synaptic and full field iGABASnFr2 traces ***I***, Ratio of peak fluorescence (Synaptic ROI/full field) for 1, 5, and 10 stimuli at 100 Hz shows a decreasing ration with stimuli LMM. Stimuli *p* = 1.4E-7. *N* = 16 imaging fields from 11 slices from three mice. *p < 0.05, ***p < 0.001.

Inhibiting GAT-1 and GAT-3 with NO-711 and SNAP-5114 had no significant effect on the Synaptic ROI but significantly increased iGABASnFR2 decay times for the full field ROI (Fig. 6*E*,*F*; LMM, Full Field ROI: Control, 242 ± 20 ms, NO-711/SNAP-5114, 400 ± 23 ms, *p* = 5.3E-7; Synaptic ROI: Control, 79 ± 6 ms, NO-711/SNAP-5114 68 ± 8 ms, *p* = 0.29). Modeling suggested that a small amount of GAT-mediated GABA clearance occurs during the timescale of an IPSC (10–30 ms). Consistent with this, there was a small, but significant, increase in iGABASnFR2 Δ*F*/*F* peak in the Synaptic ROI when GATs were inhibited (Fig. 6*G*; LMM, peak Δ*F*/*F* (%), Control: 0.11 ± 0.04, NO-711/SNAP-5114: 0.13 ± 0.04, *p* = 0.0015). For a single stimulus, the Synaptic ROI had a significantly larger peak than the full field of view. As GABA diffuses out of the Synaptic ROI, we would predict that GABA accumulates in the extracellular space and thereby reduces the difference between the peaks of the Synaptic ROI and the full field of view. As predicted, the enrichment of synaptic iGABASnFr2 peak responses, compared with full field Δ*F*/*F*, was significantly reduced with stimulus number [Fig. 6*H,I*; LMM, Stimulus *p* < 1.4 × 10^−7^, from *n* = 16 imaging fields (control and inhibitor) from 11 slices from three mice]. These results agree with computational findings and suggest that most GABA rapidly diffuses out from the synapse where it accumulates in the extrasynaptic space during repeated stimulation. Subsequently, in the extracellular space, GATs can then clear the GABA, with only small contributions in the Synaptic ROI.

### Functional consequences of rapid GABA diffusion

Modeling and confocal imaging suggest that GABA diffuses from the site of release before GATs can remove it from the extracellular space. If true, we would predict that GABA released at one synapse could spillover and modulate signaling from neighboring synapses, as well as activate extrasynaptic GABA_A_ or GABA_B_ receptors. To test the this possibility, we asked whether GABA released from one set of synapses could diffuse and induce heterosynaptic GABA_A_R desensitization of receptors activated by a second set of synapses (Overstreet et al., 2000).

Evoked IPSCs were recorded from a layer V pyramidal neuron in response to two stimulators placed 200–350 µm medially and laterally from the recorded neuron to evoke GABA release from different synaptic sites (Armbruster et al., 2016). We began by evoking an IPSC using a single stimulus to the first stimulator and quantifying its amplitude. Next, we evoked a large GABA release event on the second stimulator (five stimuli at 100 Hz) and then 100 ms later again evoked an IPSC using a single stimulus on the first stimulator. The response to the first stimulator was isolated by subtracting the preceding stimulation in isolation (Fig. 7*A*). Consistent with our hypothesis, we found that a large GABA release event evoked by preceding activity at Stimulator 2 significantly reduced the amplitude of the IPSC evoked by the first stimulator. We suspect this decrease in IPSC amplitude is driven by GABA_A_R desensitization in response to GABA released during the large event evoked by Stimulator 2. We then pharmacologically inhibited GATs, thereby enhancing GABA spillover drive by the activation of Stimulator 2, and found that this enhanced the suppression of the IPSC evoked by Stimulator 1 (NO-711 + SNAP5114; Fig. 7*B*,*C*), consistent with GATs constrains heterosynaptic inhibition (LMM, effect of prestimulus, Control IPSC: 0.87 ± 0.04, *p* = 0.038; SNAP5114/NO711: 0.73 ± 0.06, *p* = 0.014, effect of SNAP5114/N0711 vs control, *p* = 0.032, *N* = 12 cells from five mice). We then performed controls to determine if other mechanisms were also at play in suppressing IPSCs evoked by Stimulator 1. To ensure that this paradigm did not alter presynaptic GABA release, we performed two controls. First, we repeated the protocol while modulating GABA_B_Rs (antagonist CGP-54626 1 µM; agonist baclofen 10 μM; Neal and Shah, 1989; Olpe et al., 1993), which have been previously reported to drive presynaptic inhibition of GABA release. The heterosynaptic depression remained intact (effect of prestimulus CGP-54626 0.86 ± 0.03 *p* = 0.001; baclofen 0.89 ± 0.11, *p* = 0.009; Fig. 7*D*), ruling out GABA_B_-mediated suppression of evoked GABA release. Second, we repeated the experiment while quantifying GABA release with iGABASnFR1. Evoked GABA release was unaffected by preceding activity at nearby synapses, suggesting presynaptic release is unaffected and the independence of the two activated pathways (LMM, ratio 1.00 ± 0.08, *p* = 0.48. *N* = 11 slices from three mice; Fig. 7*E*,*F*). Finally, to further validate the independence of the two activated pathways, we asked if simultaneous evoked IPSCs on both stimulators were additive compared with each delivered in isolation. We found that the two synaptic inputs summated nearly linearly [ratio of peak IPSC simultaneous/individual IPSCs summated = 0.93 ± 0.06 LMM *p* = 0.45 not significantly different from 1 (perfect summation), *N* = 11 cells, three mice]. This is consistent with different synapse being activated by each stimulator. This supports our hypothesis that GABA diffuses from its site of release to drive heterosynaptic suppression of IPSCs, likely due to GABA_A_R desensitization, and that GAT activity restrains this GABA spillover and subsequent heterosynaptic suppression of inhibition.

**Figure 7.**
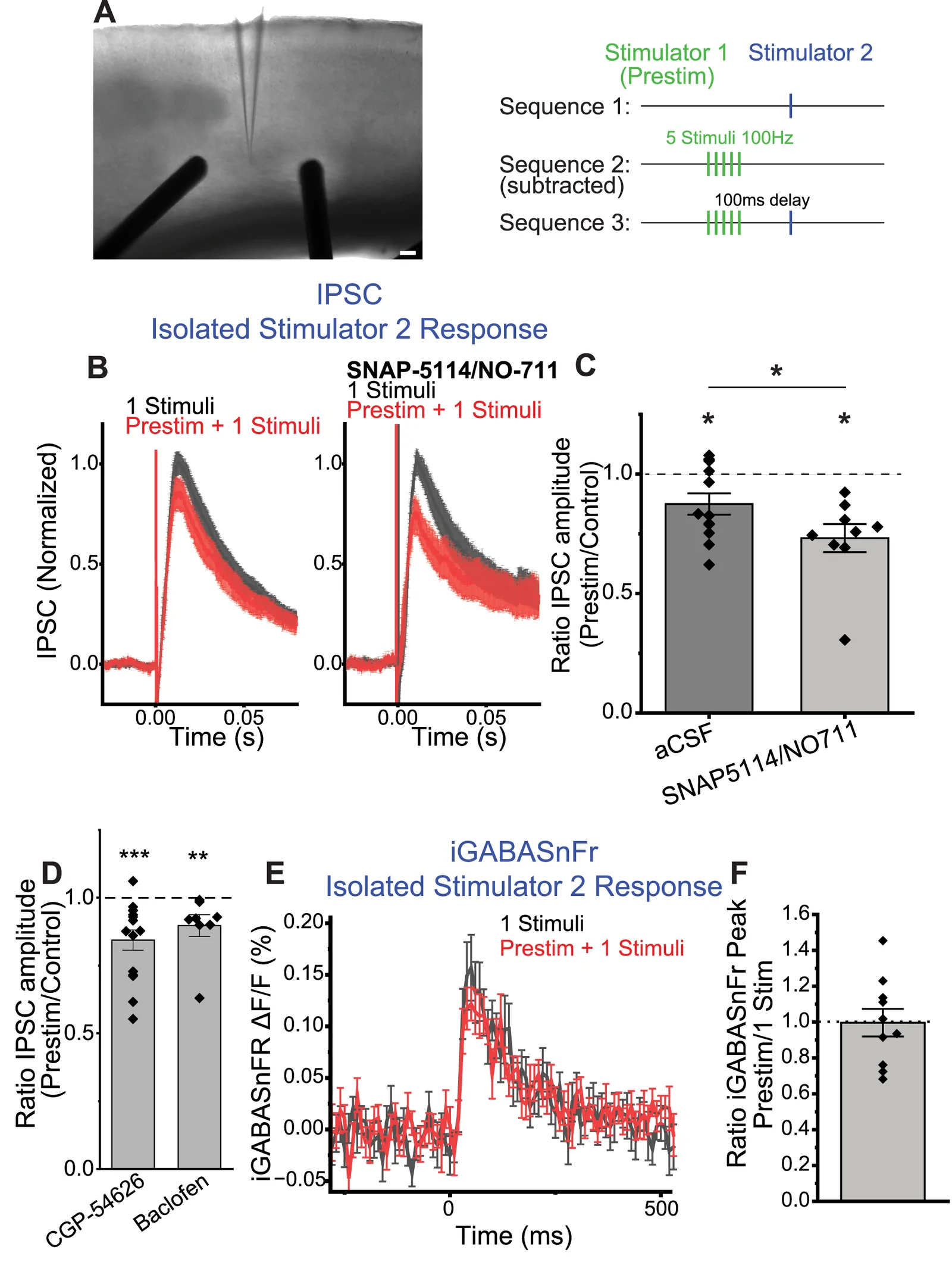
Local GABA diffusion reduces available receptors. ***A***, Two stimulators are placed laterally in layer V of the somatosensory cortex compared with the patched pyramidal neuron. On Stimulator 2, single stimuli are used to evoke IPSCs, with or without a preceding five stimuli 100 Hz train on Stimulator 1. IPSC responses to Stimulator 2 are isolated by subtracting traces. ***B***, Isolated Stimulator 2 IPSCs show reduced amplitude when there is a preceding stimulus. Inhibiting GATs with SNAP5114 and NO711 enhances the effects of the prestimulus train quantified in ***C***. LMM, effect of prestimulus train Control *p* = 0.0380, SNAP5114/NO711 *p* = 0.014, effect of SNAP5114/NO711 *p* = 0.032, *N* = 12 cells from five mice. Post hoc test paired-sample *t* test *p* = 0.03, one-sample *t* test for aCSF *p* = 0.017, SNAP5114/NO711 *p* = 0.0019. ***D***, Prestimulus effect on IPSCs was independent of GABA_B_Rs. Prestimulus effect was maintained in the GABA_B_ antagonist CGP-54626 (1 µM) and GABA_B_ agonist baclofen (10 μM), suggesting that it is not mediated by GABA_B_R LMM (*p* = 0.001, *N* = 14 cells from 3 mice CGP-54626; *p* = 0.009, *N* = 8 cells from 3 mice baclofen). ***E***, iGABASnFR1 imaging with the same paradigm shows no effect of the prestimulus train (***F***), suggesting no changes in presynaptic release. LMM *p* = 0.48. *N* = 11 slices from 3 mice. *p < 0.05, **p < 0.01.

## Discussion

Here we use iGABASnFr to quantify GABA persistence in the extracellular space following synaptic release. Our results indicate that in the somatosensory cortex, GABA transport capacity is rapidly overwhelmed, resulting in slow GABA clearance in the extracellular space following short bursts of activity. Epifluorescence imaging reveals that the persistence of GABA in the extracellular space is related to its local concentration, with slower clearance in areas of more abundant GABA release (Fig. 1). This is supported by a relatively small effect of GAT inhibition (Fig. 4), suggesting that transport capacity is overwhelmed for short bursts of activity. Modeling and confocal imaging suggest the majority of synaptically released GABA rapidly diffuses from the release site, with only a small fraction locally removed by GATs (Fig. 5). Our findings build on previous findings from GAT currents (Kinney and Spain, 2002), modeling (Overstreet and Westbrook, 2003), sniffer patches and iGABASnFr imaging of pre-ictal activity (Magloire et al., 2023; Shimoda et al., 2024), and GABA_B_ measurements (Isaacson et al., 1993) that suggest GABA clearance is slow and that GABA can significantly diffuse in the extracellular space.

There are a number of caveats to consider in our findings. First, it is critical to consider how well iGABASnFr fluorescence represents the true underlying GABA dynamics. All iGABASnFr traces represent the convolution of the sensor properties and the underlying GABA dynamics. This is especially important when considering the fast kinetics in synaptic confocal imaging or small stimuli in epifluorescence imaging, where the sensor's properties play a larger role. The slower kinetics in response to trains of stimuli should better reflect the underlying kinetics as the sensor kinetics represent a far smaller proportion of the total. In addition, our data are limited to acute brain slice preparations, but other studies have found similar slow iGABASnFr clearance kinetics in vivo (Shimoda et al., 2024; Rezaei et al., 2025). Our modeling suggests that one of the key differences between the rapid glutamate clearance and the slow GABA clearance is the abundance of the transporters, with experimental estimates indicating that GAT expression is far lower than that of glutamate transporters (Lehre and Danbolt, 1998; Chiu et al., 2002; Melone et al., 2015). A limitation of our modeling approach, however, is that it only addresses a single synapse in isolation and cannot account for the extracellular GABA accumulation from multiple synapses. Additionally, our model assumes homogeneous extracellular space and localization. Future models could incorporate more detailed extracellular geometry and transporter localization, including presynaptic GAT localization (Conti et al., 2004) and the summation of multiple GABA release sites. Extensive data suggest that GAT inhibition can modulate evoked IPSCs within their fast timescale (Rossi and Hamann, 1998; Calcagnotto et al., 2002; Overstreet and Westbrook, 2003; Keros and Hablitz, 2005) but that spontaneous, miniature, or unitary IPSCs are largely insensitive to GAT inhibition (Jensen et al., 2003). Our findings provide a potential mechanistic explanation for these divergent findings. First, our modeling replicates the miniature IPSC case of a single release site and mirrors the published data by showing only minimal effects of GATs spatially around the synapse or within the timescale of an IPSC (Fig. 2). Next, our confocal imaging shows that in areas around putative GABA release sites, GAT inhibition has minimal effects on GABA dynamics (Fig. 6). In addition, the larger role of GATs in response to evoked IPSCs suggests that multisynapse summation is critical to reveal the role of GATs. Confocal iGABASnFR imaging supports the summation of GABA in the extracellular space (Fig. 6) driving heterosynaptic GABAR desensitization that is restrained by GAT activity (Fig. 7). This suggests that while GATs have a minimal role at single release sites, they do contribute to constraining the extracellular summation and diffusion of GABA. Spatiotemporal GABA summation in the extracellular space is also consistent with our epifluorescence iGABASnFR imaging that shows that higher local GABA levels are associated with slowed clearance (Fig. 1). The clear effects of GAT inhibition on evoked IPSCs may depend on small effects observed on the peak iGABASnFr2 signal with confocal imaging and the lower affinity of synaptic GABAR (Dixon et al., 2014). Together these findings suggest that the spatial organization of inhibitory synapses and local GABA concentration is crucial to extracellular GABA dynamics, consistent with dense perisomatic synapses being more sensitive to GAT inhibition than sparse distal synapses (Gonzalez-Burgos et al., 2009).

The slow clearance and summation of GABA in the extracellular space is not reflected in IPSC recordings. Instead, IPSCs rapidly depress during trains of activity (Varela et al., 1999), likely due to a combination of GABA_A_R localization, kinetics, deactivation, and desensitization. Optical readouts of synaptic vesicle release in interneurons (Santos et al., 2013; Bae et al., 2020) have similarly showed continued GABA release during trains of activity, similar to results we report with iGABASnFr, and supporting the idea that IPSCs do not directly reflect extracellular GABA dynamics (Fig. 3). In addition, different classes of GABAergic interneurons evoke unique patterns of synaptic inhibition. PV-positive interneurons drive rapidly suppressing synaptic inhibition, while inhibition from somatostatin-positive interneurons is facilitated during bursts of activity (Ma et al., 2012). This raises the possibility that due to our stimulation protocol and underlying differential postsynaptic targeting, pyramidal cell IPSCs may preferentially reflect PV-cell activity, while iGABASnFr data may reflect a combination of PV and somatostatin-mediated GABA release. This raises new questions about the relationship of interneuron activity, especially during high-frequency action potential firing, extracellular GABA dynamics, and the evoked phasic synaptic inhibition. In addition, our data suggest that during bursts of GABA release, the distinction between phasic and tonic inhibition can be rapidly blurred. Specifically, during bursts of GABA release, phasic signaling rapidly depresses but extracellular GABA accumulates based on the local spatiotemporal pattern of activity. A similar idea has been proposed for neurogliaform interneurons, which provide a spatially broad GABA signal, in line with volume transmission (Overstreet-Wadiche and McBain, 2015), and have distinctly slow IPSCs that likely engage GABAR across large areas. Similarly, GABAergic spillover in the cerebellum has been suggested to cause small amplitude slow IPSCs via high-affinity α6-containing GABA_A_Rs (Rossi and Hamann, 1998). Lastly, a recent study using GABA sniffer patches and iGABASnFr shows a volumetric GABA signal preceding epileptiform discharges (Magloire et al., 2023). Our results would suggest a similar effect with bursts of activity; however, unlike with neurogliaform interneurons which have distinct slow IPSCs, equivalent slow IPSCs due to volumetric signaling have not been identified in the cortex. Our data also suggest that this extracellular GABA summation could lead to heterosynaptic desensitization of GABAR and could dynamically modulate tonic GABA-mediated inhibition.

Critical for interpreting these results is understanding how the evoked activity reflects physiological activity in vivo. PV interneuron peak firing rates can easily reach over 100 Hz, while somatostatin interneurons have lower firing rates. Additionally, interneurons show some synchronous bursting (Puig et al., 2008; Voloh and Womelsdorf, 2018), suggesting that our stimulus paradigms generally align within physiological activity ranges. Our model system of fluorescence imaging of acute brain slices biases the activity toward more shallow depth in the slice; however, previous work with iGluSnFr has shown a strong correlation with neuronal NMDA receptor currents. The spatially broad activation and imaging modalities have made it difficult to directly characterize GABA diffusion; instead we have focused on the persistence of GABA in the extracellular space as it is more directly tied to downstream physiological effects. Lastly, the interface at the brain-slice–aCSF boundary potentially enables for diffusional escape of GABA. This could potentially minimize observed activity that occurs on a slower timescale; however, this should not mask faster; this would however still show slow GAT activity. These studies and our findings imply that, even for a single stimulus, GABA diffuses and rapidly transitions from local phasic inhibition to tonic inhibition.

In summary, we use iGABASnFR imaging to identify novel and unexpected aspects of extracellular GABA dynamics that have important implications on synaptic integration, information processing, and the metabolic cost of sustaining GABA release during heightened activity. Our findings also suggest that synaptically released GABA rapidly diffuses to drive extracellular GABA summation that is much more dynamic and spatially heterogeneous than previously appreciated. Our findings raise important questions about how GABAergic inhibition, which relies on fast-spiking neurons to drive temporal precision, can operate in a context of slow, spatially broad extracellular neurotransmission, suggesting we have much to learn about the fundamentals of inhibitory neurotransmission.
